# The Angiotensin II Type 1 Receptor-Associated Protein Attenuates Angiotensin II-Mediated Inhibition of the Renal Outer Medullary Potassium Channel in Collecting Duct Cells

**DOI:** 10.3389/fphys.2021.642409

**Published:** 2021-05-14

**Authors:** Juliano Zequini Polidoro, Nancy Amaral Rebouças, Adriana Castello Costa Girardi

**Affiliations:** ^1^Heart Institute (InCor), University of São Paulo Medical School, São Paulo, Brazil; ^2^Department of Physiology and Biophysics, Institute of Biomedical Sciences, University of São Paulo, São Paulo, Brazil

**Keywords:** K^+^ homeostasis, K^+^ channels, angiotensin II type 1 receptor, c-Src, angiotensin II type 1 receptor-associated protein, kidney

## Abstract

Adjustments in renal K^+^ excretion constitute a central mechanism for K^+^ homeostasis. The renal outer medullary potassium (ROMK) channel accounts for the major K^+^ secretory route in collecting ducts during basal conditions. Activation of the angiotensin II (Ang II) type 1 receptor (AT1R) by Ang II is known to inhibit ROMK activity under the setting of K^+^ dietary restriction, underscoring the role of the AT1R in K^+^ conservation. The present study aimed to investigate whether an AT1R binding partner, the AT1R-associated protein (ATRAP), impacts Ang II-mediated ROMK regulation in collecting duct cells and, if so, to gain insight into the potential underlying mechanisms. To this end, we overexpressed either ATRAP or β-galactosidase (LacZ; used as a control), in M-1 cells, a model line of cortical collecting duct cells. We then assessed ROMK channel activity by employing a novel fluorescence-based microplate assay. Experiments were performed in the presence of 10^−10^ M Ang II or vehicle for 40 min. We observed that Ang II-induced a significant inhibition of ROMK in LacZ, but not in ATRAP-overexpressed M-1 cells. Inhibition of ROMK-mediated K^+^ secretion by Ang II was accompanied by lower ROMK cell surface expression. Conversely, Ang II did not affect the ROMK-cell surface abundance in M-1 cells transfected with ATRAP. Additionally, diminished response to Ang II in M-1 cells overexpressing ATRAP was accompanied by decreased c-Src phosphorylation at the tyrosine 416. Unexpectedly, reduced phospho-c-Src levels were also found in M-1 cells, overexpressing ATRAP treated with vehicle, suggesting that ATRAP can also downregulate this kinase independently of Ang II-AT1R activation. Collectively, our data support that ATRAP attenuates inhibition of ROMK by Ang II in collecting duct cells, presumably by reducing c-Src activation and blocking ROMK internalization. The potential role of ATRAP in K^+^ homeostasis and/or disorders awaits further investigation.

## Introduction

Appropriate regulation of K^+^ content in the extracellular fluid is critical for cell function, especially for excitable tissues, since the K^+^ gradient largely determines resting membrane potential (Unwin et al., 2011). Accordingly, many different acute and chronic mechanisms are now recognized to play a role in K^+^ homeostasis (Mcdonough and Youn, 2017) in such a way that blood K^+^ levels tend to vary slightly throughout the day (Moore-Ede, 1986). The task of maintaining short‐ and long-term K^+^ balance is mainly achieved by kidneys, which properly regulate K^+^ content in urine in response to the physiological status. Indeed, kaliuresis can be as high as ~150% or as low as ~2% of total filtered potassium (Unwin et al., 2011). By doing so, kidneys can respond to critical situations of ion excess or restriction.

Seminal studies by Giebisch, Malnic, and Klose during the 1960s have shown the crucial role of distal nephron to the regulated kaliuresis under different experimental conditions (Malnic et al., 1964). They have found that the proximal tubule and the Henle of Loop segments reabsorb ~90% of all filtered K^+^, regardless of the amount of K^+^ contained in the diet. On the other hand, the appropriate regulation of renal losses occurs in the downstream segments by further K^+^ reabsorption or secretion from the peritubular space (Malnic et al., 1964).

The renal outer medullary potassium (ROMK) channel comprises the major constitutive pathway for renal K^+^ secretion (Wang and Giebisch, 2009; Welling and Ho, 2009), thereby playing an essential role in K^+^ homeostasis. Under the setting of K^+^ restriction, K^+^ homeostasis can be maintained at least in part by inhibition of ROMK in the collecting duct *via* coupling of angiotensin II (Ang II) to its Ang II type 1 receptor (AT1R; Wei et al., 2007). Ang II/AT-1R induces oxidative stress and enhances the levels of total and phosphorylated c-Src, which ultimately inhibits ROMK by its phosphorylation at a tyrosine residue (Wei et al., 2007; Yue et al., 2011). Accordingly, AT1R is locally upregulated in collecting ducts during low K^+^-diets (LK; Wei et al., 2007), and AT1R inhibition during LK diets impairs renal K^+^ conservation (Jin et al., 2009), underlining the importance of renin-angiotensin-system for K^+^ homeostasis.

Several studies have pointed out that AT1R signaling is modulated by associated proteins, particularly the AT1R-associated protein (ATRAP). ATRAP colocalizes *in vivo* with AT1R in all nephron segments, from the Bowman’s capsule to the inner medullary collecting duct cells (Tsurumi et al., 2006). It has been shown that ATRAP is often an inhibitory partner of AT1R by, at least in part, impairing G-protein coupling to the receptor (Lopez-Ilasaca et al., 2003). However, it has also been reported that ATRAP can potentiate, instead of inhibiting, AT1R-mediated effects (Barro-Soria et al., 2012) and that ATRAP may also induce AT1R-independent actions (Mederle et al., 2016). The impact of ATRAP on AT1R/Ang II-mediated sodium homeostasis has been previously shown (Wakui et al., 2013; Ohsawa et al., 2014).

However, the potential role of ATRAP on Ang II-regulation of K^+^ homeostasis remains to be established. Therefore, the present study aimed to test the hypothesis that ATRAP impacts Ang II-mediated ROMK regulation and if so, to gain insight into the potential underlying mechanisms.

## Materials and Methods

### Reagents and Antibodies

Reagents were obtained from ThermoFisher Scientific (Rockford, IL, United States) unless otherwise noted. VU591, a specific ROMK inhibitor (Bhave et al., 2011), and dexamethasone were acquired from Sigma-Aldrich (St. Louis, MO, United States). Ang II was purchased from Tocris Bioscience (Bristol, United Kingdom). Anti-α-epithelial sodium channel (ENaC; C-20), anti-β-ENaC (E-10), and anti-γ-ENaC (H-110) antibodies were obtained from Santa Cruz Biotechnology (Santa Cruz, CA, United States). Anti-ROMK (SAB2501215), anti-V5 (R960-25), and anti-phospho-c-Src (#2101) antibodies were acquired from Sigma-Aldrich, ThermoFisher Scientific, and Cell Signaling (Beverly, MA, United States), respectively. Anti-GAPDH antibodies (Ab8245 and sc-20357) were obtained from Abcam and Santa Cruz Biotechnology. Horseradish peroxidase-conjugated antibodies were obtained from Jackson ImmunoResearch (West Grove, PA, United States).

### Cell Culture

**Figure 1:**
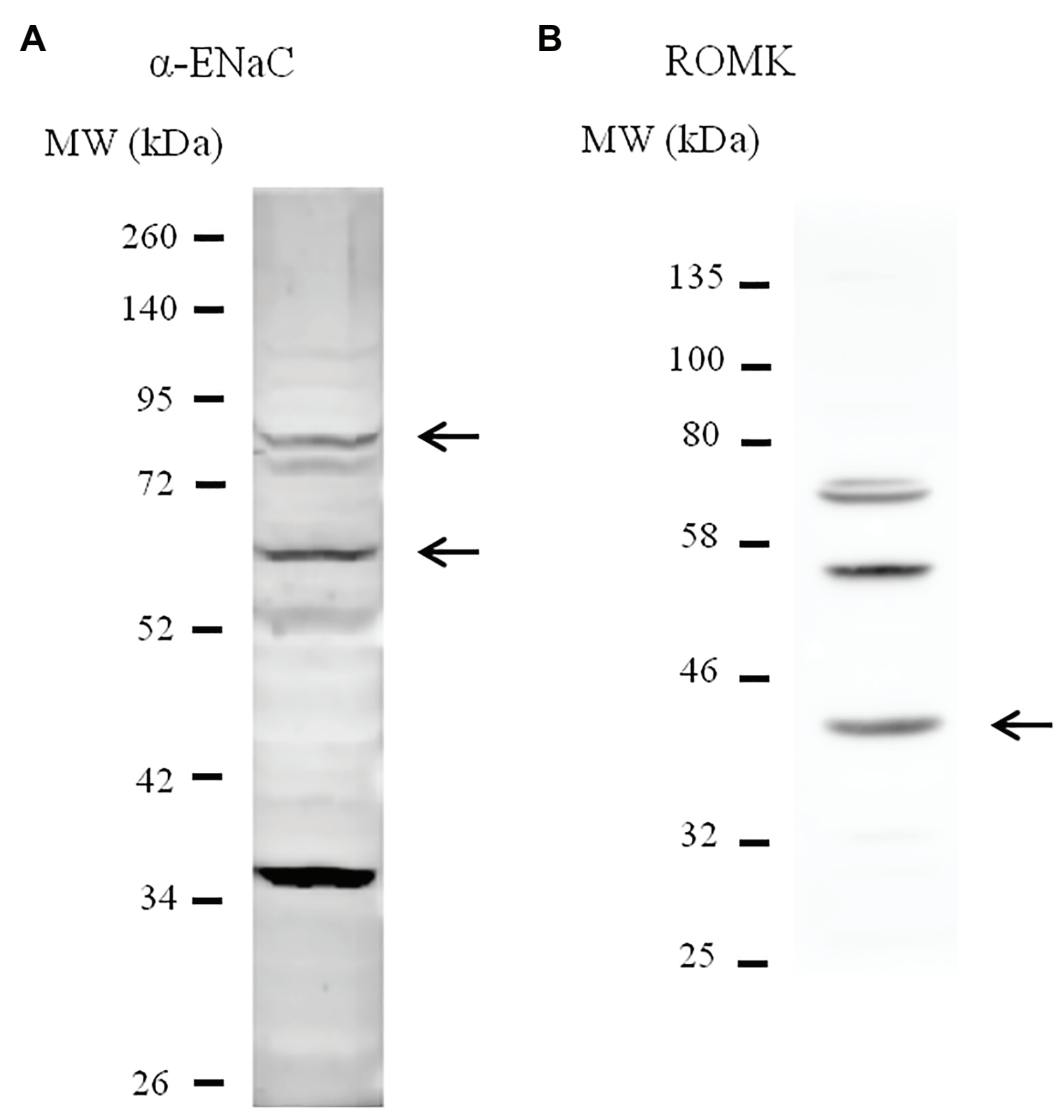
Characterization of M-1 cell line by immunoblotting. Lysates from M-1 cells were immunoblotted with antibodies against anti-α-epithelial sodium channel (ENaC; **A**)and anti-renal outer medullary potassium (ROMK; **B**) for cell line characterization. Arrows indicate the predicted bands that represent each protein, including full-length and the cleaved form of α-ENaC.

M-1 collecting duct cells (ATCC CRL-2038) were kindly provided by Dr. Per Svenningsen (University of South Denmark, Denmark) and maintained in 25-cm^2^ tissue culture flasks in a 1:1 mixture of Dulbecco’s modified Eagle’s medium/Ham’s F12 medium (DMEM/F12) supplemented by 100 U/ml penicillin, 100 μg/ml streptomycin, 0.5 mM sodium pyruvate, 5 μM dexamethasone, and 5% (v/v) heat-inactivated fetal bovine serum (FBS) as recommended by the supplier. Cultures were incubated in a humidified 95% air-5% CO_2_ atmosphere at 37°C and subcultured by 0.25% trypsin-EDTA. Cells were seeded onto 24-well microplates for immunoblotting experiments or 96-well microplates for FluxOR assay experiments. Non-transfected cells were grown to confluence and then serum-starved for at least 72 h. Lysates from M-1 cells were immunoblotted with antibodies against the α subunit of ENaC and ROMK for cell line characterization (Figures 1A,B). Band sizes were predicted based on previous evidence (Hu et al., 2009; Murthy et al., 2016; Shimizu et al., 2017).

### ATRAP Overexpression in M-1 Cells

M-1 ATRAP coding sequence was amplified using 5' primer and 3' primer containing Hind III and Not I restriction sequences, respectively (5'-ACACAAGCTTGGGATGGAGCTGCCTGCCGTGAAC-3' and 5'-ACACGCGGCC-GCGGTACCCCCGGGGGGCAGCTTG-3') and cloned into pcDNA6/V5-His vector (ThermoFisher). pAcGFP1-C1 (Clontech) was used to assess lipotransfection efficiency. Cultures were serum-deprived at ~90% confluence and lipotransfected with pcDNA6/V5-His/galactosidase (LacZ) control vector, encoding a β-galactosidase protein, or with pcDNA6/V5-His/ATRAP vector, both of them tagged to V5-His at their C-termini. For every 2 cm^2^ of cell culture, 2 μg of plasmid DNA and 1-2 μl lipofectamine 2000 were added to the serum-free medium for 24 h. Cells were then maintained in a serum-free medium for at least more 48 h before conducting functional experiments.

### FluxOR Assay

Renal outer medullary potassium channel activity was assessed by a fluorescence-based microplate assay (FluxOR II Green Potassium Ion Channel Assay). The assay is based on the fact that thallium influx serves as a surrogate for K^+^ channel and transporter activity and takes advantage of recently developed thallium-sensitive fluorophores (Beacham et al., 2010). The FluxOR assay was performed using the washing method recommended by the supplier, and bottom fluorescence intensity was assessed by end-point readout in the SynergyH1 microplate reader (Biotek, Winooski, VT).

### SDS-PAGE and Immunoblotting

M-1 cells were solubilized by radioimmunoprecipitation assay (RIPA) buffer (140 mM NaCl, 1% Triton X-100, 0.1% sodium deoxycholate, 0.1% sodium dodecyl sulfate (SDS), and 10 mM Tris-HCl pH 8.0), supplemented with 5 mM EDTA, 1 mM EGTA, 0.2 mM PMSF, and protease cocktails [cOmplete, Mini, EDTA-free protease inhibitor cocktail (Roche) or Protease Inhibitor Cocktail P8340 (Sigma-Aldrich)]. Phosphatases inhibitor cocktails [P5726 and P0044 (Sigma-Aldrich)], at 1:150 dilution, were also added to RIPA buffer when assessing phospho-c-Src expression. Protein supernatants were obtained by centrifuging cell lysates at 14,000 rpm for 20 min. Protein quantification was performed using the BCA or Lowry assay. The appropriate amount of lysate was denatured by Laemmli buffer (2% SDS, 20% glycerol, 100 mM β-mercaptoethanol, and 0.1% bromophenol blue, and 50 mM Tris, pH 6.8). About 7.5–12%, SDS polyacrylamide gel electrophoresis (SDS-PAGE) was performed as described previously (Crajoinas et al., 2016). Proteins were transferred to polyvinylidene difluoride (PVDF) membranes (Millipore Immobilon-P, Bedford, MA) by the semi-dry system [Owl HEP-1 (ThermoFisher)] for 3 h at 400 mA or by wet transfer systems [TE 62 (GE Healthcare, Little Chalfont, United Kingdom)] overnight at 350 mA, 4°C. Western-immunoblotting experiments using V5 and phospho-c-Src antibodies were performed following the supplier’s recommendations. For the remaining antibodies, PVDF membranes were blocked with TBST solution (5% nonfat dry milk and 0.1% Tween 20 in Tris-Buffered Saline (TBS), pH 7.4) for 1 h, and then incubated with the appropriate primary antibody overnight. Primary antibody dilutions were 1:500 for anti-ROMK, 1:1,000 for anti-phospho-c-Src, 1:1,000 for sc-20357, and 1:5,000 for Ab8245 and anti-V5. Membranes were then washed five times for 10 min and incubated with the respective secondary antibody at 1:2,000 dilution for 1 h. Densitometry analyses of digitized bands were performed by ImageJ software (National Institutes of Health, Bethesda, MD, United States).

### Cell Surface Biotinylation

Cell surface biotinylation was performed to assess ROMK expression in the plasma membrane, as previously described (Carraro-Lacroix et al., 2010). LacZ‐ and ATRAP-overexpressing M-1 cells cultivated in six-well plates were treated with 10^−10^ M Ang II for 40 min before biotinylation protocol as previously described in detail (Carneiro De Morais et al., 2015). Biotinylated cells were then lysed by a modified RIPA buffer (150 mM NaCl, 50 mM Tris·HCl, 1% Triton X-100, 0.5% sodium deoxycholate, and 5 mM EDTA, pH 7.4) and 300 μg of the total cellular extract were incubated with 50 μl streptavidin-coupled agarose beads (#20347, ThermoFisher). After overnight incubation, streptavidin beads were washed with RIPA buffer for 15 min, followed by centrifugation at 10.000 rpm for 5 min. This washing step was repeated three times. Biotinylated proteins were then denatured from streptavidin beads by adding modified Laemmli buffer (2% SDS, 20% glycerol, 100 mM DTT, 0.1% bromophenol blue, 50 mM Tris, and pH 6.8) and heating samples at 98°C for 3 min. Solubilized biotinylated proteins were submitted to SDS-PAGE. Finally, we have also used 10 μg of the total cellular extract from each sample to assess total ROMK and GAPDH expression.

### Statistical Analysis

Data are presented as mean ± SE. Statistical analyses were performed using GraphPad Prism 7.04. Groups were tested for normality by the Shapiro-Wilk test and homoscedasticity by the Brown-Forsythe test. When comparing three or more groups, we used a parametric ANOVA test followed by Bonferroni *post hoc* test or nonparametric Kruskal-Wallis followed by Dunn’s *post hoc* test. For comparison between two groups, we used an unpaired *t*-test.

## Results

### Ang II Inhibits ROMK Activity in M-1 Cells

**Figure 2:**
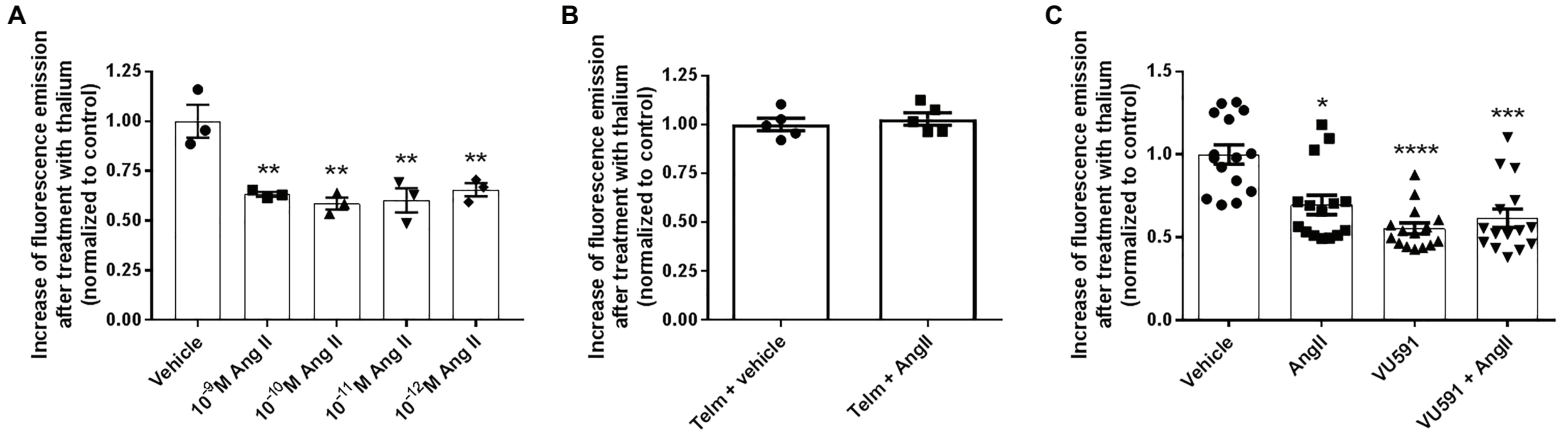
Angiotensin II (Ang II) inhibits ROMK-mediated K^+^ conductance in M-1 cells. **(A)** M-1 cells were treated with different Ang II concentrations or vehicle for 30 min (*n* = 3). ^**^p < 0.01 vs. vehicle-treated M-1 cells. The *p* value was calculated using ANOVA followed by the Bonferroni *post hoc* test. **(B)** M-1 cells were pretreated with telmisartan for 60 min and then treated with vehicle or Ang II (10^−10^ M) for 40 min. **(C)** M-1 cells were treated with Ang II (10^−10^ M) or vehicle in the presence or absence of the selective ROMK inhibitor VU591 (10 μM) for 40 min (*n* = 15). ^*^p<0.05; ^***^p<0.001; ^****^p<0.0001 vs. vehicle-treated M-1 cells. The *p* value was calculated by using the Kruskal-Wallis test, followed by Dunn’s *post hoc* test.

To assess whether K^+^ transport in M-1 cells was responsive to Ang II, we employed the FluxOR assay, as described in Materials and Methods. Experiments were conducted in the presence or absence of the ROMK inhibitor VU591. As seen in Figure 2A, the K^+^ conductance in non-transfected M-1 cells, measured as the thallium-induced fluorescence, was similarly reduced by Ang II at concentrations ranging from 10^−9^ to 10^−12^ M. The concentration of 10^−10^ M Ang II was chosen to be used in the subsequent experiments, an average urinary concentration of Ang II (Wang et al., 2003).

In order to assess whether the Ang II-mediated effect on K^+^ conductance was induced by AT1R activation, M-1 cells were then pretreated with telmisartan (10^−8^ M) before exposing cells to vehicle or Ang II (10^−10^ M). As observed in Figure 2B, telmisartan pretreatment abrogates Ang II-induced inhibition of K^+^ conductance. Finally, M-1 cells were also treated with Ang II or vehicle for 40 min in the presence or absence of 10 μM VU591. As illustrated in Figure 2C, the VU591 compound reduced K^+^ conductance by ~45 ± 3% in comparison with vehicle-treated cells, underlining the critical role of the ROMK channel in apical K^+^ conductance in M-1 cells. Similar to the findings of Figure 2A, Ang II decreased K^+^ conductance by ~30 ± 6% compared to vehicle-treated M-1 cells. Importantly, VU591 and Ang II cotreatment did not induce an additive inhibitory effect on K^+^ conductance, supporting the notion that Ang II-mediated modulation of K^+^ transport in M-1 cells is due to the regulation of ROMK activity.

### ATRAP Overexpression Attenuates the Ang II Inhibitory Effect on ROMK

**Figure 3:**
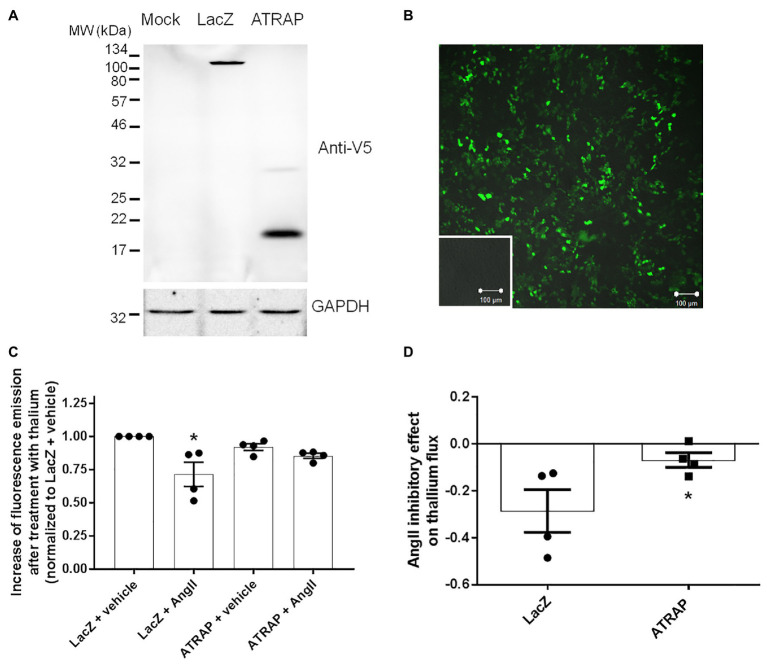
Angiotensin II type 1 receptor-associated protein (ATRAP)-overexpression attenuates Ang II-mediated ROMK inhibition in M-1 cells. **(A)** Representative immunoblot of M-1 cells transfected with galactosidase (LacZ-V5) and ATRAP-V5 vectors probed with an anti-V5 antibody. **(B)** Evaluation of lipotransfection protocol efficiency in M-1 cells that were transfected with a GFP-encoding vector. Green fluorescence emission in M-1 cells was detected using a 505–550 nm filter in a confocal microscope after exciting GFP-transfected and mock-transfected M-1 cells at 488 nm laser light. Images were captured at 10x magnification. Mock-transfected M-1 cells are displayed in the inset. **(C)** K^+^ conductance was determined as the increase of fluorescence emission after thallium incubation in M-1 cells overexpressing LacZ or ATRAP treated with 10^−10^ M Ang II or vehicle for 40 min (*n* = 4). ^*^p < 0.05 vs. vehicle-treated LacZ M-1 cells. The *p* value was calculated by the Kruskal-Wallis test, followed by Dunn’s *post hoc* test. **(D)** K^+^ conductance inhibition by Ang II treatment in LacZ‐ and ATRAP-overexpressing M-1 cells. ^*^p < 0.05 vs. LacZ-overexpressing M-1 cells, using unpaired *t*-test.

The efficiency of M-1 transfection was evaluated by the emission of fluorescence in mock‐ or GFP-transfected M-1 cells and the efficacy of overexpression of ATRAP and recombinant β-LacZ was confirmed by immunoblotting using an anti-V5 antibody (Figures 3A,B). We then sought to examine the effect of overexpressing ATRAP in the Ang II-mediated ROMK inhibition in M-1 cells. As seen in Figure 3C, Ang II significantly reduced ROMK activity in control LacZ-M-1 cells (~29 ± 9%, *p* < 0.05), but not in ATRAP-overexpressing cells (~15 ± 2%), when compared to vehicle-treated LacZ-overexpressing M-1 cells.

Interestingly, a significant part of this discrete inhibitory effect in K^+^ conductance observed in ATRAP-overexpressing M-1 cells is present at baseline conditions (8 ± 3% inhibition vs. vehicle-treated LacZ-overexpressing cells). In fact, when this difference at basal levels is taken into account, the magnitude of ROMK inhibition mediated by Ang II in ATRAP-overexpressing cells is only 7 ± 3% when compared to vehicle-treated ATRAP-overexpressing cells, an effect significantly smaller than that observed in LacZ-overexpressing cells (Figure 3D). These data collectively suggest that ATRAP overexpression attenuates Ang II-inhibition of ROMK but does not increase K^+^ conductance at basal conditions.

### ATRAP Overexpression Blunts Ang II-Induced Decrease of Cell Surface ROMK Expression

**Figure 4:**
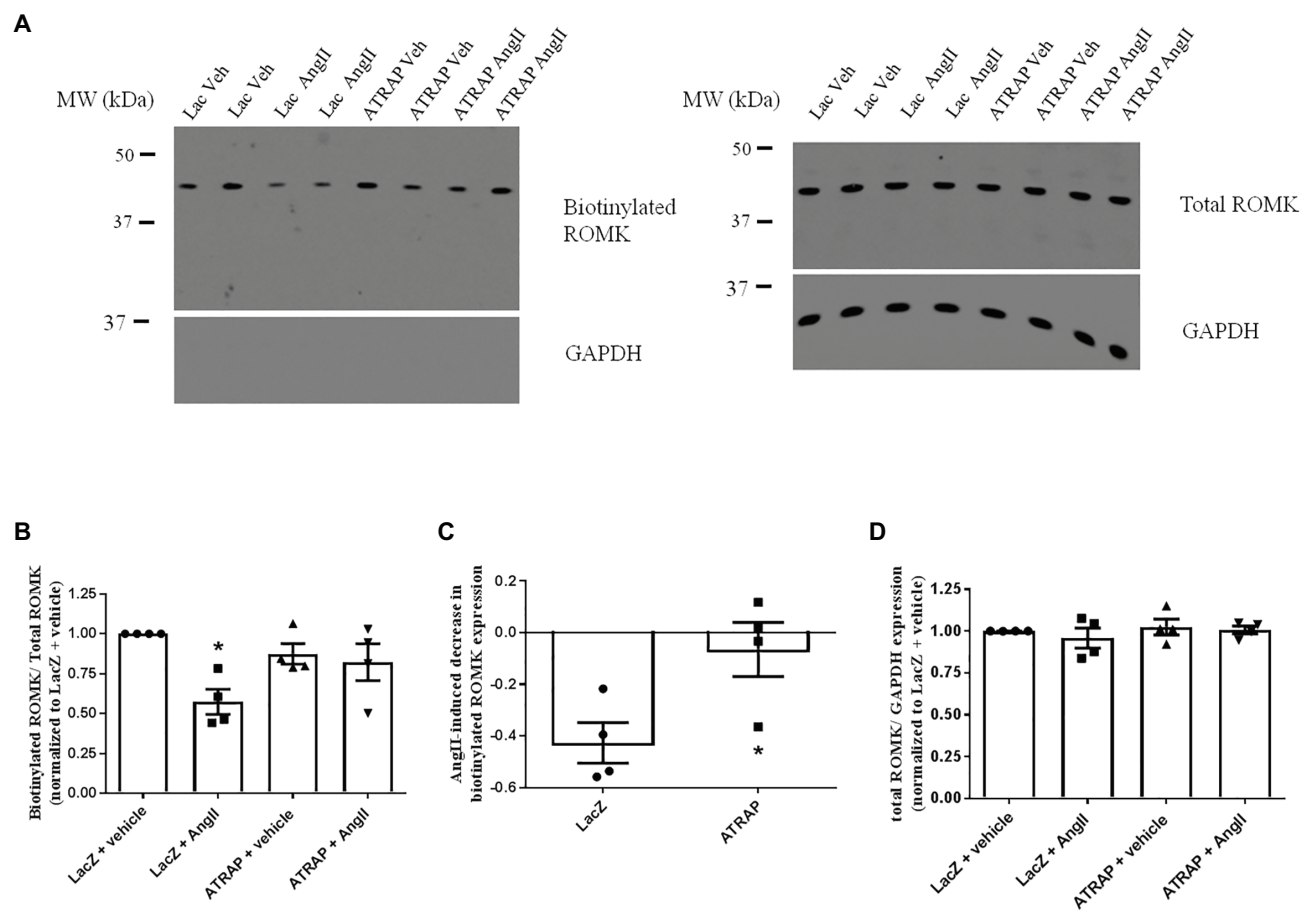
AT1R-associated protein-overexpression blunts Ang II-mediated decrease of plasma membrane ROMK expression. M-1 cells overexpressing LacZ-V5 or ATRAP-V5 were treated with vehicle or 10^−10^ M Ang II for 30–40 min before assessing plasma membrane ROMK expression by biotinylation protocol. **(A)** Representative immunoblot image of biotinylated ROMK, total ROMK, and GAPDH. GAPDH was also used as a control to assess purity in biotinylated samples (Supplementary Material). **(B)** Densitometry analysis of bands from biotinylated ROMK (~45-kda) normalized to total ROMK expression (*n* = 4). As biotinylation protocol was performed in six-well plates, band intensities were normalized to each plate’s respective control (vehicle-treated LacZ sample). ^*^p < 0.05 vs. vehicle-treated LacZ-overexpressing M-1 cells, using Kruskal-Wallis test followed by Dunn’s *post hoc* test. **(C)** Ang II-mediated reduction of ROMK expression at the plasma membrane in LacZ‐ and ATRAP-overexpressing M1-cells. ^*^p < 0.05 vs. LacZ-overexpressing M-1 cells, using unpaired *t*-test. **(D)** Densitometry analysis of bands from total ROMK normalized to GAPDH expression (*n* = 4).

To assess whether the decrease of Ang II-induced inhibition of ROMK in ATRAP-overexpressing cells was associated with a reduction in ROMK expression at the plasma membrane, we evaluated ROMK expression plasma membrane by cell surface biotinylation. As presented in Figures 4A,B, after acute treatment with Ang II, Lac-Z overexpressing cells, but not ATRAP-overexpressing cells, showed a significant decrease in the ratio of surface ROMK to total ROMK expression, when compared to vehicle-treated LacZ-overexpressing cells (43 ± 8%, *p* < 0.05). It was observed that Ang II treatment caused a reduction of ROMK cell surface expression in only one out of four samples from ATRAP-overexpressing cells. As seen in Figure 4C, the mean Ang II-induced effect on reducing plasma membrane ROMK expression was just 6 ± 10% in the ATRAP-overexpressing group (*p* < 0.05 vs. LacZ-overexpressing cells). Finally, we also assessed total ROMK expression normalized to GAPDH expression, and no difference was observed among experimental groups (Figure 4D).

### ATRAP Attenuates Activation of c-Src in the Presence and Absence of Ang II

**Figure 5:**
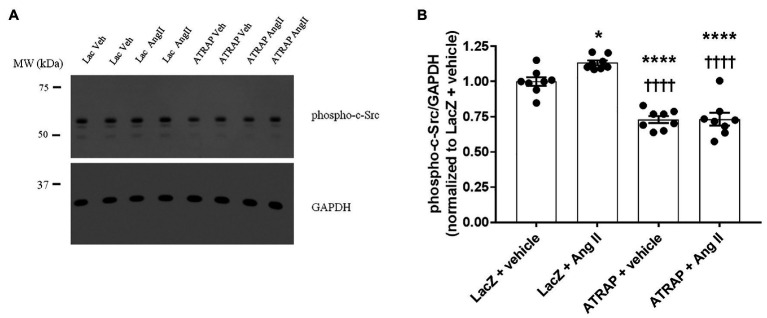
The effect of Ang II on the levels of phospho-c-Src in radioimmunoprecipitation assay (RIPA)-soluble lysates from M-1 cells overexpressing LacZ and ATRAP protein. M-1 cells overexpressing LacZ-V5 and ATRAP-V5 were acutely treated with vehicle or 10^−10^ M Ang II for 15 min and assessed for c-Src phosphorylation status. **(A)** Representative immunoblot of phospho-c-Src (Supplementary Material). **(B)** Graphical representation of the ratio of phosphorylated Src to GAPDH (*n* = 8). ^*^p < 0.05; ^****^p < 0.0001 vs. vehicle-treated LacZ-M-1 cells. ^††††^*p* < 0.0001 vs. Ang II-treated-LacZ-M-1 cells. All statistical analyses were performed using ANOVA, followed by Bonferroni *post hoc* test.

The activation of the c-Src kinase by phosphorylation of the tyrosine 416 residue constitutes the central molecular mechanism of ROMK inhibition by Ang II (Yue et al., 2011). Thus, we next assessed whether modulation of c-Src activation could underlie the diminished response to Ang II in M-1 cells overexpressing ATRAP compared with LacZ‐ overexpressing M-1 cells. The representative immunoblot of phospho-c-Src in ATRAP and Lac-Z-overexpressed M-1 cells treated or not with 10-^10^ M Ang II is shown in Figure 5A. The phospho-c-Src levels were significantly elevated in LacZ-overexpressing M-1 cells treated with Ang II vs. vehicle-treated LacZ-overexpressing M-1 cells (Figures 5A,B). In contrast, phosphorylation of c-Src at tyrosine 416 significantly decreased in both vehicle‐ and Ang II-treated ATRAP-overexpressing M-1 cells compared to LacZ-overexpressing M-1 cells (Figures 5A,B).

## Discussion

Understanding kaliuresis regulation is crucial since plasma K^+^ levels need to be kept within a narrow range for vital cellular functions. As the median K^+^ consumption in modern western diets is ~50% of the recommended intake (Chen et al., 2006), understanding how kidneys are able to decrease kaliuresis is particularly important. It is well established that AT1R upregulation in collecting duct cells with consequent ROMK inhibition constitutes an essential mechanism for K^+^ conservation during low-K^+^ diets. Indeed, it has been demonstrated that rats fed a low K^+^ diet and simultaneously treated with losartan do not exhibit a decrease in the number of ROMK-like channels in their collecting ducts and exhibit a higher reduction in plasma potassium levels during potassium restriction (Jin et al., 2009). Our study provides new evidence that the angiotensin receptor-associated protein ATRAP impacts K^+^ conductance in collecting duct cells. More specifically, ATRAP counteracts the Ang II-mediated ROMK internalization and consequent inhibition in a collecting cell culture model. This effect is accompanied by decreases in c-Src Y416 phosphorylation, a critical signaling pathway for ROMK inhibition by Ang II.

Although p38 and ERK1/2 pathways seem to be part of AT1R-dependent inhibition of ROMK channels during LK diets (Babilonia et al., 2006; Jin et al., 2009), tyrosine phosphorylation of ROMK by c-Src is the most important signaling pathway directly responsible for ROMK inhibition during potassium restriction. While collecting ducts from rats fed LK diets do not show relevant ROMK-like small conductance K^+^ currents, these same collecting ducts presented a significant increase in the number of ROMK-like channels in patch-clamp recordings when the collecting duct cells were acutely treated with c-Src inhibitor herbimicyn A for 30 min, an effect that does not occur in rats receiving HK diets (Wang et al., 2000, 2010). Also, in the collecting ducts from animals receiving LK diets, the acute treatment with c-Src inhibitor herbymicin A majorly abolishes the Ang II inhibitory effect over ROMK activity (Wei et al., 2007). Accordingly, in the present study, diminished response to Ang II in M-1 cells overexpressing ATRAP was accompanied by a decrease in c-Src phosphorylation at the tyrosine 416. Interestingly, we have observed lower activation of c-Src even in the vehicle-treated ATRAP-M1 cells, suggesting that ATRAP not only inhibits Ang II-mediated c-Src signaling but also evokes Ang II/AT1R-independent modulation of this kinase. It has been previously shown that ATRAP can regulate signaling pathways in the absence of Ang II. Interestingly, ATRAP interacts with the multiscaffolding RACK1 (Wang et al., 2002), a protein that is also detected in AT1R immunocomplexes (Parent et al., 2008) and is associated with c-Src sequestration (Kiely et al., 2005). Since RACK1 is not an integral membrane protein, ATRAP might recruit RACK1 to the plasma membrane, which would favor c-Src inactivation, even without Ang II involvement.

Albeit ATRAP overexpression *per se* decreased c-Src phosphorylation, we did not observe increased ROMK activity in the vehicle-treated ATRAP overexpressed-M1 cells. This finding could be expected considering the effect of c-Src inhibition by herbymicin A in collecting duct cells from animals fed an LK diet. Other AT1R-independent effects mediated by ATRAP or cellular compensatory mechanisms might take place in such a way that K^+^ channel activity does not change in cells overexpressing ATRAP despite the reduction in c-Src activation. This observation resembles what was observed for the ATRAP-mediated effect on cardiomyocyte Ca^2+^ signaling (Mederle et al., 2016). The authors found that ATRAP stimulates SERCA2a and accelerates calcium sequestration into the endoplasmic reticulum *in vitro*, which improves ventricular relaxing and filling. However, ATRAP knockout cells also had an unexpectedly lower basal intracellular Ca^2+^ concentration, which led the authors to postulate that ATRAP could regulate other calcium homeostasis mechanisms at basal conditions. When these basal differences were not considered, the ATRAP effect over physiological relevant calcium transients was somewhat masked. Accordingly, when we take into account the modest basal variations of K^+^ conductance between LacZ and ATRAP-overexpressing cells, the inhibitory effect of Ang II on ROMK is even lower in the ATRAP-overexpressing M-1 cells (7% inhibition in ATRAP-overexpressing group, in comparison to a 29% inhibitory effect of Ang II in LacZ-overexpressing cells).

It is known that ROMK inhibition by c-Src is not directly caused by channel phosphorylation. Previous studies demonstrated that ROMK inhibition by exogenous c-Src was only seen in cell-attached patches but not in isolated patches. This inhibitory effect was associated with phosphorylation of tyrosine 337 of the channel and was abolished by agents that inhibit endocytosis (Wang et al., 2000; Moral et al., 2001). Therefore, ROMK inhibition by c-Src is mediated by phosphorylation and consequent internalization of the channel. In line with these observations, we observed that Ang II treatment induced a significant decrease in plasma membrane expression of ROMK in LacZ-overexpressing cells, but not in ATRAP-overexpressing cells, and the level of changes observed in ROMK activity assay somehow resembles what we have observed in biotinylation experiments. It is worth mentioning that previous studies assessing ROMK inhibition by AT1R/Ang II have deduced ROMK internalization by single-channel patch-clamp experiments and further analysis of NPo products (Wei et al., 2007; Jin et al., 2009; Wang et al., 2010). Therefore, the present study provides novel molecular evidence that Ang II reduces ROMK cell surface expression in collecting duct cells.

Whereas the ATRAP’s impact on sodium balance and volemic homeostasis has been shown in different studies, particularly during Ang II-induced hypertension (Ohsawa et al., 2014), its implications for kaliuresis regulation are unknown. K^+^ conservation through ROMK inhibition is an essential mechanism for K^+^ homeostasis without evoking other electrolyte imbalances. When such a regulatory mechanism is not enough to maintain K^+^ balance during LK diets, the consequent decreases of extracellular K^+^ levels induce hyperactivation of NCC transporter, thus linking K^+^ restriction to hypertension (Terker et al., 2015). Therefore, it is possible that differential regulation of ROMK by Ang II/AT1R and ATRAP in collecting ducts that we found here may have consequences in serum K^+^ levels and could impact not only K^+^ homeostasis but also pressure outcomes in K^+^-restricted diets. In this regard, Yap et al. (2017) have observed that the polymorphism A1166C at the 3'-UTR of the AT1R gene correlates with alterations in blood pressure that seems dependent on K^+^ intake in healthy Malaysian adults. Subjects with the genotype AA, associated with lower expression of AT1R (Sethupathy et al., 2007; Chandra et al., 2014), showed higher systolic and diastolic blood pressure when their K^+^ intakes were low. Also, He et al. (2011) observed that this genotype was also associated with higher blood pressure decline after potassium supplementation in Chinese subjects. It remains to be established whether such changes in blood pressure during dietary K^+^ restriction may also be associated with genetic variations in other RAS components, including ATRAP.

The impact of differential regulation of ATRAP on ROMK regulation and, consequently, on K^+^ homeostasis and blood pressure control could resemble some effects of losartan treatment observed during poor-K^+^ diets (Jin et al., 2009). As aforementioned, losartan treatment increases kaliuresis and exacerbates hypokalemia during the LK diet in rats (Jin et al., 2009). This condition could also upregulate NCC activity and, by doing so, attenuate the natriuretic effect of such a drug. Interestingly, the K^+^-rich DASH diet amplifies losartan treatment response, particularly in African-American subjects, so that the combined treatment is more than additive (Conlin et al., 2003). It is known that African-Americans represent a population that has diminished blood pressure response to AT1 receptor blockers and, at the same time, increased blood pressure response to thiazides (Brewster and Seedat, 2013). Notably, a genetic study reported that, for African-American women, such increased thiazide response was associated with the genotype AA of the polymorphism A1166C (Frazier et al., 2004). Taking these studies into account and considering ATRAP effects on ROMK regulation, the classical hypotensive effect of ATRAP reported in normal-K^+^ diets could be blunt during diets with a modest or more severe K^+^ restriction.

On the other hand, during HK diets, ATRAP might be an important component determining the K^+^ secretion capacity of collecting ducts. Interestingly, Wei et al. (2014) observed that CCDs from rats submitted to HK rats and pretreated with AT2R antagonists did not show a decreased ROMK activity after AngII treatment. This observation suggests that collecting duct AT1R during HK settings is not responsive to modulate ROMK activity. Notably, AT1R expression, assessed at the transcriptional level, was not decreased during the HK diet. It would be interesting to evaluate further whether ATRAP is upregulated in CCDs during HK diets, which might explain these previous observations made by Wei and colleagues. Although our *in vitro* observations give us direct evidence of ATRAP’s ability to attenuate AT1R-mediated ROMK inhibition, further *in vivo* works will be important for assessing how ATRAP is modulated during changes in potassium intake and how CCD-specific ATRAP transgenic animals respond to potassium dietary changes, for instance. These analyses will address ATRAP’s physiological impact on potassium balance and might help us to explain previous physiological observations in this field.

**Figure 6:**
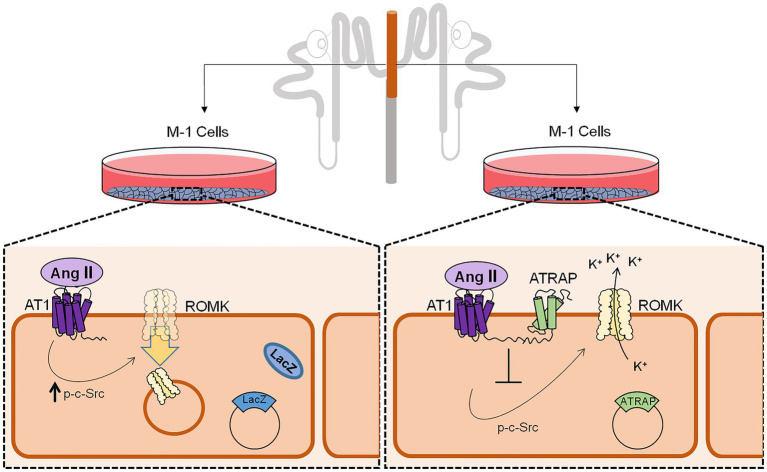
Schematic representation of ATRAP-mediated actions on c-Src phosphorylation status, ROMK expression at the plasma membrane, and ROMK activity. M-1 cells overexpressing control LacZ protein has a decreased K^+^ conductance after Ang II treatment, an effect that was associated with lower expression of ROMK at the plasma membrane and induction of c-Src phosphorylation, the classical signaling pathway for ROMK inhibition by Ang II. Conversely, M-1 cells overexpressing the ATRAP do not show significant decreases in K^+^ conductance after Ang II treatment and plasma membrane ROMK expression, which was also associated with a blunted response of c-Src phosphorylation.

In conclusion, our study demonstrates that ATRAP can counteract Ang II-mediated inhibition of ROMK activity and might then be a relevant protein regulating K^+^ handling. As depicted in Figure 6, K^+^ conductance is inhibited in LacZ-overexpressing M-1 cells treated with Ang II. In contrast, this response is blunted in M1 cells overexpressing ATRAP. Accordingly, this differential response is accompanied by a decrease in the expression of ROMK channels at the plasma membrane of LacZ-overexpressing M-1 cells, but not in ATRAP-overexpressing cells. Finally, blunted response to Ang II in ATRAP-overexpressing M1 cells is associated with diminished c-Src phosphorylation signaling, a pathway that seems to be critically involved in ROMK endocytosis. Future studies should be performed in animal models to evaluate the impact of differential ATRAP expression in K^+^ homeostasis during different changes of K^+^ dietary content. Moreover, it would be interesting to assess how polymorphisms in ATRAP could be associated with changes in plasma K^+^ levels and/or in blood pressure during K^+^-poor diets in different human populations.

## Data Availability Statement

The raw data supporting the conclusions of this article will be made available by the authors, without undue reservation.

## Author Contributions

AG and NR designed the study. JP carried out the experiments, analyzed the data, wrote the manuscript, and prepared the figures. All authors contributed to the article and approved the submitted version.

### Conflict of Interest

The authors declare that the research was conducted in the absence of any commercial or financial relationships that could be construed as a potential conflict of interest.
