# Supplementary material for: The Angiotensin II Type 1 Receptor-Associated Protein Attenuates Angiotensin II-Mediated Inhibition of the Renal Outer Medullary Potassium Channel in Collecting Duct Cells
Source: Front Physiol. 2021 May 14;12:642409. doi: 10.3389/fphys.2021.642409 (PMC8160308; doi:10.3389/fphys.2021.642409)
Supplement: Supplementary file 1 [file Data_Sheet_1.docx]

Supplementary Material

**The AT1R-associated protein (ATRAP) attenuates angiotensin II-mediated inhibition of the ROMK channel in collecting duct cells**

**Juliano Z. Polidoro^1^, Nancy A. Rebouças^2^; Adriana C. C. Girardi^1^***

^1^Heart Institute (InCor) University of São Paulo Medical School, São Paulo, SP, Brazil

^2^Department of Physiology and Biophysics, Institute of Biomedical Sciences, University of São Paulo, São Paulo, SP, Brazil

**Supplementary Figure S1: Full unedited gels - Figure 4**

**
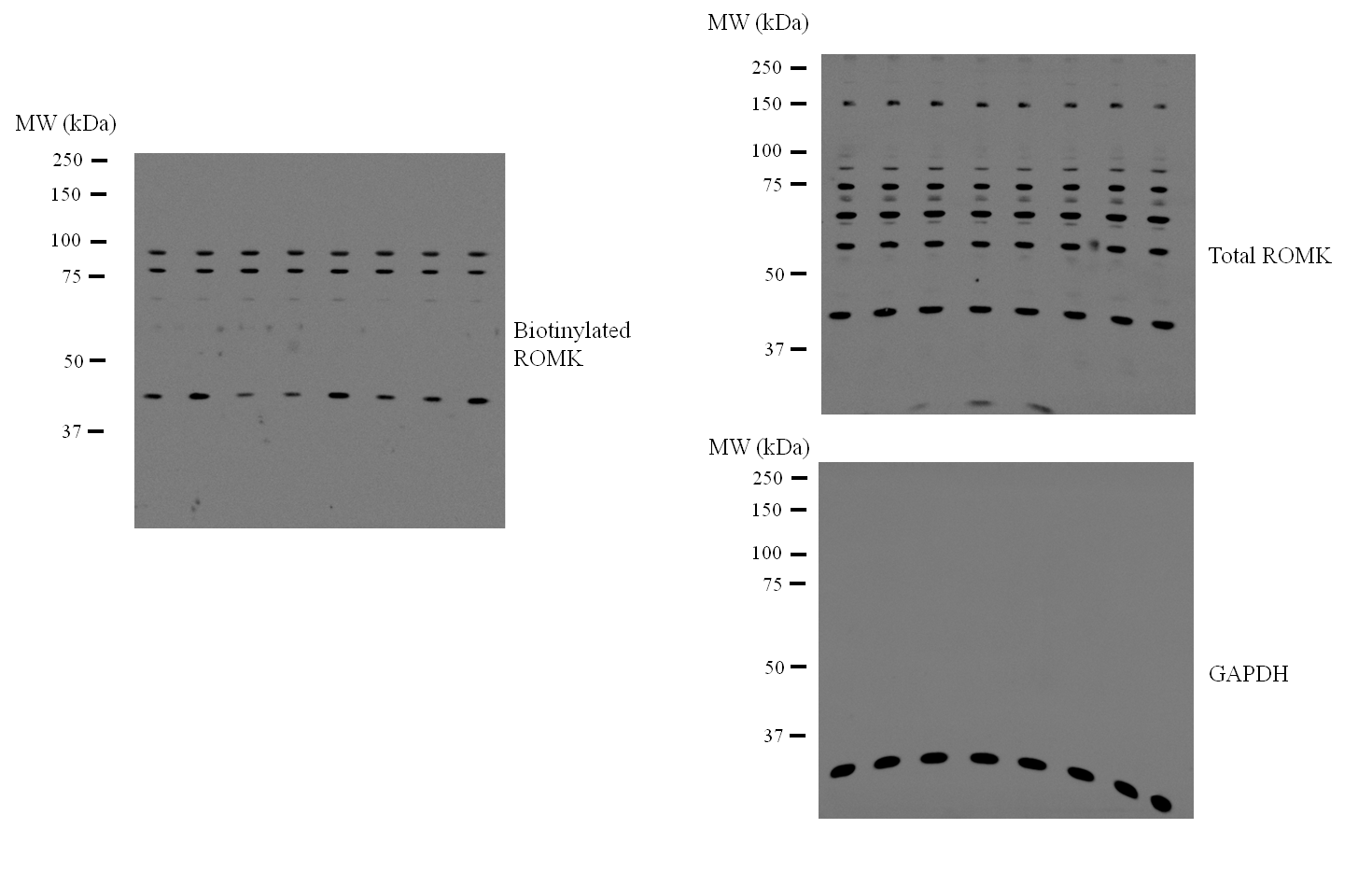
**

**Supplementary Figure S1: Full unedited gels - Figure 5**

**
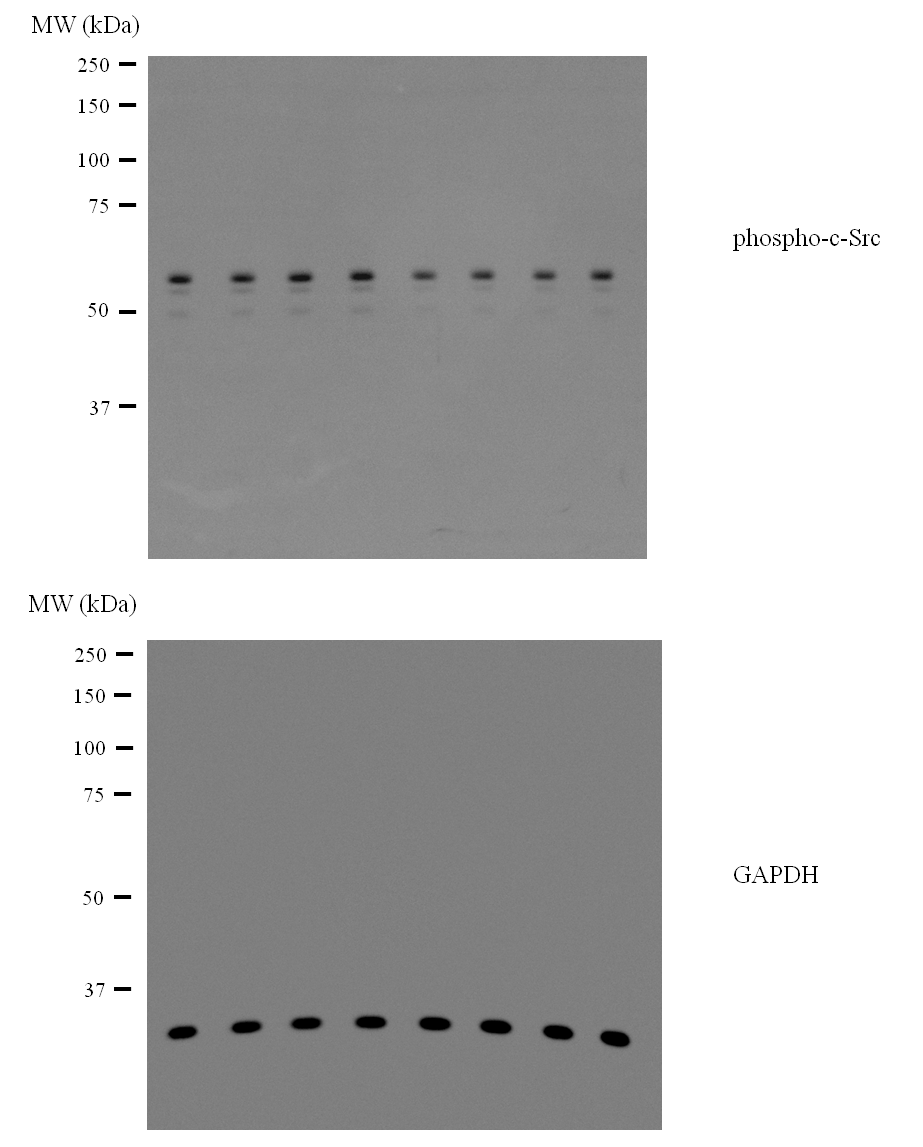
**
